# *Ex vivo* expansion of cord blood-derived endothelial cells using a novel xeno-free culture media

**DOI:** 10.2144/fsoa-2018-0103

**Published:** 2019-03-22

**Authors:** Ayokunle A Ogunye, Ian K McNiece, Sharon Daniliuc, Mira Genser-Nir, Yuliya-Yael Miropolski, David Fiorentini, Joshua N Kellner

**Affiliations:** 1 Department of Stem Cell Transplantation & Cellular Therapy, The University of Texas MD Anderson Cancer Center, Houston, TX, 77030 USA; 2 Biological Industries, Kibbutz Beit-Haemek, Israel

**Keywords:** cell therapy, cord blood, endothelial cells, endothelial progenitor cell, *ex vivo* expansion

## Abstract

**Aim:**

Endothelial cells (ECs), isolated from peripheral blood (PB), bone marrow (BM) and cord blood (CB), are limited in numbers and expansion has had limited success. We used a novel serum-free medium (EndoGo) to evaluate effects on *ex vivo* expansion of CB-derived ECs.

**Materials & methods:**

Flow cytometry and matrigel were used to determine expansion of ECs and for determination of the EC progenitor cell.

**Results:**

EndoGo™-containing cultures demonstrated superior expansion and stimulated proliferation of two distinct subpopulations, CD34^+^CD31^+^ and CD34^-^CD31^+^, which exhibited different morphology, phenotype and function. EndoGo also expanded the CB endothelial progenitor cells from freshly isolated CB.

**Conclusion:**

These findings demonstrate the potential of EndoGo to expand CB ECs, which could generate increased numbers of ECs for therapeutic applications.

## Lay abstract

Endothelial cells are important for the development of blood and lymph vessels, needed for proper blood and lymph system function. Damage to these vessels can occur with various conditions and diseases. Many tissues cannot regenerate, requiring therapy. Cell therapy can be an important tool in accelerating the repair and recovery process. Development of a cell expansion protocol unique to endothelial cells, initiated from a widely available source as in cord blood, is important to achieve this process. We demonstrate a unique medium that can promote proliferation of endothelial cells that could be further developed for clinical settings.

Endothelial cells (ECs) contribute to the formation of the vasculature in both normal and pathological processes through either angiogenesis, that is, sprouting growth from established vasculature, or through vasculogenesis, the new assembly of blood vessels [1]. These processes are initiated through endothelial progenitor cells (EPCs) that exist in the bone marrow (BM) and mobilize to the site of neovascularization [2]. This mobilization has been observed during ischemic events, wound healing and tumor growth [3–5]. Attempts to promote mobilization through exogenous methods have been explored; however, the low frequency of circulating EPCs and further damage via indirect mechanisms has limited this approach [6,7]. Infusion of EPCs through cellular therapy may be more effective in treating and preventing disease.

EPCs have also recently become a focus for regenerative medicine, as use in cellular therapy could treat a number of different conditions, including ischemia [8], heart disease [9], stroke [10] and diabetes [11]. In fact, many clinical trials treating various diseases have been attempted using ECs from BM and peripheral blood (PB) with varied success or inconclusive findings [12]. Asahara *et al*. first identified the EPC in PB and have further provided the baseline in understanding the progenitor phenotype [13]. Although various groups have identified EPCs from different sources, including BM, PB and cord blood (CB), isolation was paired with limited frequency and mixed phenotypes [14–17]. Although CD133, CD34 and VEGFR2 have been shown to be expressed by EPCs, this phenotype is not unique to this population. Hematopoietic stem cells (HSCs) also maintain an identical phenotype [18]. Analogous markers and functional characteristics have been established with PB monocytes further confounding the study of EPCs [19]. Establishing a unique phenotype for EPCs would facilitate better characterization of the biology and development of ECs and enable development of *ex vivo* expansion protocols.

*Ex vivo* expansion of hematopoietic cells has been used in clinical trials in applications aimed at enhancing hematopoietic engraftment [20]. Many of the clinical trials attempted to date have involved isolation of mononuclear cells (MNCs) from BM or mobilized PB for collections of EPCs, with inconclusive results regarding the success of EPC involvement (reviewed in [12]). Attempts to isolate and expand EPCs *in vitro* have been successful in preclinical experiments but are insufficient in yielding the numbers of cells needed for effective clinical applications [8,21]. Reports suggesting clinical scale expansion have been achieved through population doubling calculations using serially passaged cultures and not with large-scale expansion [22,23]. *In vitro* cultures have enabled identification of two types of ECs, termed early-outgrowth and late-outgrowth [24]. Early EPCs have typically resembled a heterogeneous population with expression of hematopoietic and myeloid markers [21,25], CD45 and CD14 respectively, while exhibiting silenced EC promoters [26]. The low frequency of early EPCs, however, has prevented more detailed analyses. Late-outgrowth cells or endothelial colony-forming cells (ECFCs) are derived after 14 days of culture and exhibit mature EC markers, although loss of progenitor markers occurs [14,24,27]. Most studies suggest that the early-EPCs support angiogenesis while the late-outgrowth may contribute primarily to capillary formation [24,28,29]. Development of new *in vitro* culture methods to expand either of these populations would enable testing the efficiency of these populations in treating various diseases or promoting angiogenesis.

In the present study, we attempted to isolate and expand EC lines from CB for potential clinical therapies. We obtained a novel cell culture medium (EndoGo XF), which we have demonstrated to enhance the expansion of ECFCs from CB. This media specifically expanded the CD34^+^ population from which CB EC lines were isolated. We further report a phenotype of the CB EPC using cell sorting and discovered unique expansion of the CB EPC and ECFC with EndoGo.

## Materials & methods

### Umbilical cord blood & isolation of CB ECs

Human umbilical CB was obtained with informed consent under The University of Texas M.D. Anderson Cancer Center Institutional Review Board (IRB)-approved protocol. CB MNCs were obtained by layering CB over Histopaque and collecting the buffy coat.

#### CB ECFC/ECs

CD45^+^, CD45^-^CD34^+^ and CD45^-^CD34^-^ cells were obtained through magnetic separation by selecting CB MNCs with CD45 microbeads and further selection of the negative fraction with CD34 microbeads (Miltenyi Biotec, CA, USA) following manufacturer's protocols. Cells were placed into 25 cm^2^ flasks in endothelial cell media (ECM) and maintained in a 37°C incubator with 5% CO_2_. Nonadherent cells and medium were harvested, pelleted and fresh media was added weekly until emergence of the adherent population was visible. After 3 weeks, CB ECs emerged only from the CD45^-^CD34^+^ fraction. Assays in this study utilized EC cell lines obtained from various CB using CD45^-^CD34^+^ selection and established with ECM. CB ECFCs and ECs were harvested with 0.05% trypsin-EDTA (Gibco BRL, NY, USA) to be either expanded or cryopreserved.

#### CB EC progenitor

CBMNCs were stained with CD45 microbeads (Miltenyi Biotec) and selected through magnetic separation columns according to manufacturer's protocols. CD45^-^ MNCs were stained with CD34, CD31, CD144, CD146, CD42a and sorted using a MoFlo Astrios (Beckman Coulter, CA, USA). Sorted populations were placed into ECM medium and medium was changed weekly until growth was observed. Antibodies were obtained from either BD Biosciences (CA, USA) or eBioscience (CA, USA).

### Endothelial cell medium

#### Endothelial cell media (ECM)

α-Minimum essential medium (α-MEM; Mediatech, Inc., VA, USA) supplemented with 20% fetal bovine serum (FBS; Akron Biotech, FL, USA), 1% penicillin–streptomycinæ Glutamine (GPS, Gibco), 20 ng/ml bFGF, 10 ng/ml VEGF and 10 ng/ml EGF. All growth factors were obtained from Peprotech (NJ, USA).

#### EndoGo

EndoGo XF was supplied by Biological Industries (CT, USA). Supplement Mix was added to EndoGo medium. Full EndoGo medium was then supplemented with 10% Human Platelet Lysate (HPL; Mill Creek Life Sciences, MN, USA) and 1% Penicillin–Streptomycin–Glutamine (GPS, Gibco). ECFCs/ECs cultured in EndoGo were removed using recombinant Trypsin Solution which was inhibited using Trypsin inhibitor (both from Biological Industries) according to manufacturer's recommendations. Due to the clotting reactivity when combining serum and EndoGo-HPL with trypsin, all cells cultured with EndoGo were washed and assayed with phosphate-buffered saline (PBS, Gibco) + 2% HPL.

#### Media test

CB ECFCs/ECs were added to various media for expansion: ECM; αMEM + 10% HPL + 20 ng/ml bFGF + 10 ng/ml VEGF +10 ng/ml EGF; EndoGo XF + 20% FBS; EndoGo + 10% FBS and EndoGo. Cells were harvested after 7 days in culture, counted and immunophenotyped for CD45, CD34 and CD31. All growth factors (EGF, VEGF, bFGF and SCF) were obtained from Peprotech (NJ, USA).

### EC flow cytometry

ECFCs/ECs were stained with fluorochrome-conjugated antibodies CD45, CD117, CD184 (BD Biosciences), CD34, CD31, CD144, CD146, CD42a (eBioscience) and CD133 (Miltenyi Biotec). Cells were prepared as follows: FACS buffer (PBS supplemented with 2% FBS) for cells in ECM or PBS supplemented with 2% HPL for cells cultured in EndoGo. Appropriate nonreactive isotype-matched fluorochrome conjugated antibodies were used for controls. Cells were acquired on either a FACSCalibur flow cytometer (BD Biosciences) using CellQuest software or a LSR Fortessa (BD Biosciences) using Diva software and analyzed by FlowJo (TreeStar, OR, USA).

### Growth time course

CB culture derived ECFCs/ECs were stained with CD34 and CD31 antibodies and sorted using a MoFlo Astrios (Beckman Coulter). Whole CB ECs and each sorted cell subpopulation, CD31^+^ CD34^+^ cells and CD31^+^CD34^-^ cells were seeded in duplicate at 2 × 10^4^ in ECM or EndoGo. Cells were harvested with trypsin-EDTA for cells in ECM or recombinant trypsin with trypsin inhibitor for cells in EndoGo and counted on day 7. Harvested cells were stained with CD34, CD45 and CD31 for flow cytometric analysis.

### Cytospin/growth slide

CB ECFCs/ECs were affixed either by direct cytospin (1600 rpm for 5 min at room temperature) or cultured in ECM for 24 hr in a 37°C, 5% CO_2_ incubator and subsequently fixed and stained. Slides were fixed and stained with Hema3 (Fisher Scientific, NH, USA). Images were obtained using Olympus DP-10 camera (Olympus Imaging, PA, USA).

### Matrigel assay

The matrigel assay was performed using Matrigel (BD Biosciences) following manufacturer's recommendations. Briefly, 200 μl Matrigel solution was added to the well and incubated at 37°C for 30 min before plating cells. Cells were plated at 30,000–40,000/well of a 96-well flat bottom plate at 100 μl volume. ECs or admixed ECs were prepared together before plating onto Matrigel.

### Statistics

Data were analyzed for statistical significance using the Student's t-test. Data and statistics were performed using GraphPad Prism 6 software (GraphPad, CA, USA).

## Results

### Growth of ECs *in vitro*

#### Ex vivo expansion of CB ECFCs/ECs

**Figure 1.**
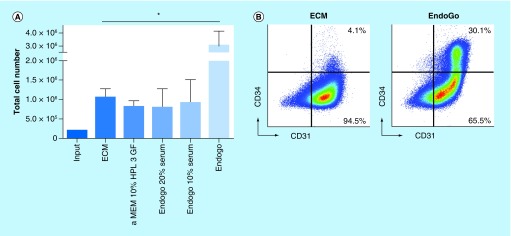

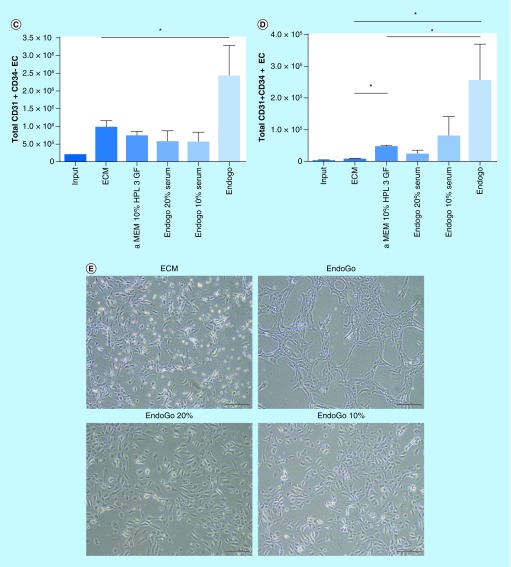
**EndoGo significantly expands cord blood ECFCs/ECs *in vitro*.** **(A)** Total cell number of ECFCs/ECs after 7-day expansion in various culture media: **(1)** ECM, **(2)** α-MEM + 10% HPL + 20 ng/ml BFGF + 10 ng/ml VEGF + 10 ng/ml EGF, **(3)** EndoGo + supplements + 20% FBS, **(4)** EndoGo + supplements + 10% FBS and **(5)** EndoGo + supplements + 10% HPL. **(B)** Representative flow cytometric plots of ECs in ECM and EndoGo. **(C)** Quantitative analysis of Day 7 CB EC CD31^+^CD34^-^ population. **(D)** Quantitative analysis of day 7 CB EC CD31^+^CD34^+^ population. **(E)** Microscopic images of day 7 CB EC cultures in ECM, EndoGo, EndoGo 20% serum and EndoGo 10% serum. In all experiments, results are shown as mean ± standard error of the mean (*p < 0.05); n = 5. CB: Cord blood; EC: Epithelial cell; ECM: Endothelial cell media; FBS: Fetal bovine serum; HPL: Human platelet lysate.

Development of clinical EC cellular therapy protocols has been limited largely due to the lack of efficient *ex vivo* EC expansion with published reports either expressing sufficient clinical levels of ECs via theoretical calculations or lacking complete phenotypic and functional study of the end populations. Numerous groups have attempted and reported EC expansion *in vitro* using different culture media; however, total EC and EPC proliferation have not been properly studied and translation into clinical protocols has not been demonstrated [8,13,21,27,30]. Since CB has become more readily available, we used this blood source and isolated CD34^+^ cells and cultured in a standard EC medium (ECM), which is similar in composition to other commercially available EC mediums. From initial seeding, CB CD34^+^ cells required 2–3 weeks before adherent cell populations were readily visible (data not shown). To determine the effectiveness of ECM in promoting CB EC expansion, we seeded 200,000 cells and cultured for 7 days in ECM (Figure 1A). ECM-induced fivefold expansion of CB ECFCs/ECs (CD45^-^CD31^+^) (1.06 ± 0.22 × 10^6^). Since our goal was to develop or optimize *ex vivo* expansion protocols of ECs, we also tested various combinations of VEGF, EGF, bFGF1 and SCF to ECM but all were suboptimal unless combined in total medium (ECM; data not shown). The addition of SCF did not promote further total cell expansion yet preserved the CD31^+^CD34^+^ population which suggests a positive role of SCF on EC progenitors (data not shown). Overall, the lack of significant cell expansion with ECM is suboptimal in promoting EPC expansion and thus prevents subsequent proliferation of the total EC population and potential clinical translation.

We obtained EndoGo XF (EndoGo; Biological Industries), a novel xeno-free EC medium, to determine the ability to expand CB ECs compared with ECM. As seen in Figure 1A, EndoGo promoted significantly greater total nucleated cell (TNC) expansion compared with ECM (3.04 ± 1.08 × 10^6^ and 1.06 ± 0.22 × 10^6^, respectively). When serum was substituted for HPL in EndoGo, TNC proliferation was inhibited (EndoGo 3.04 ± 1.08 × 10^6^; 10% FBS, 0.92 ± 0.592 × 10^6^; 20% FBS, 0.802 ± 0.48 × 10^6^). Alternatively, substitution of HPL for FBS in ECM (aMEM 10% HPL 3GF) did not improve cell expansion when compared with ECM (0.82 ± 0.15 × 10^6^ and 1.06 ± 0.22 × 10^6^, respectively).

#### Phenotype & morphology of *ex vivo* expanded CB ECs

Since ECs are phenotypically defined from blood sources using CD45 and CD31 expression and ECs were established from CD34 selected CB, we used this panel to phenotype the *ex vivo* expanded progeny. Figure 1B shows the representative flow phenotype of CB ECFCs/ECs expanded with ECM and EndoGo. Cells were first gated on CD45, of which CB ECs were contained in the CD45 negative fraction (99–100%). Although CD31^+^CD34^-^ ECs were significantly expanded with EndoGo (ECM- 0.96 ± 0.2 × 10^6^, EndoGo- 2.4 ± 0.29 × 10^6^; Figure 1C), the expansion of the CD31^+^CD34^+^ population was most pronounced in EndoGo when compared with ECM (0.25 ± 0.1 × 10^6^ and 6004 ± 2915, respectively) (Figure 1D). This suggests that EndoGo might interact specifically with the progenitor cell population (CBMNC CD45^-^CD34^+^) to induce both proliferation (of total CD31^+^ ECs) and self-renewal of EPCs (of total CD34^+^ cells). Of note was the increase in CD31^+^CD34^+^ ECs from the addition of 10% HPL to α-MEM medium with EGF, bFGF and VEGF compared with ECM, suggesting some effect on the EPC though not sufficient to expand the population similar to EndoGo (α-MEM 10%-HPL: 46000 ± 4000 and ECM: 6004 ± 2915). The morphology of *in vitro* CB ECFCs/ECs cultured with different media also varied (Figure 1E). Although ECM promoted more fibroblastic, tube-like cells, the addition of serum to EndoGo produced smaller, circular cells that spread out in culture. EndoGo promoted fibroblast-like cells that formed tube-like structures during *in vitro* culture. It is unclear if growth of ECs in different *in vitro* cultures promotes expansion of subtypes of ECs that we did not phenotype or if media is altering the attachment of ECs to the culture flask. Overall, this suggests that EndoGo, including platelet lysate and supplement, is superior for optimal expansion of CB ECs.

### Phenotypic & functional characteristics of CB ECs expanded in EndoGo

#### Flow characterization of EndoGo expanded CB ECFCs/ECs

**Figure 2.**
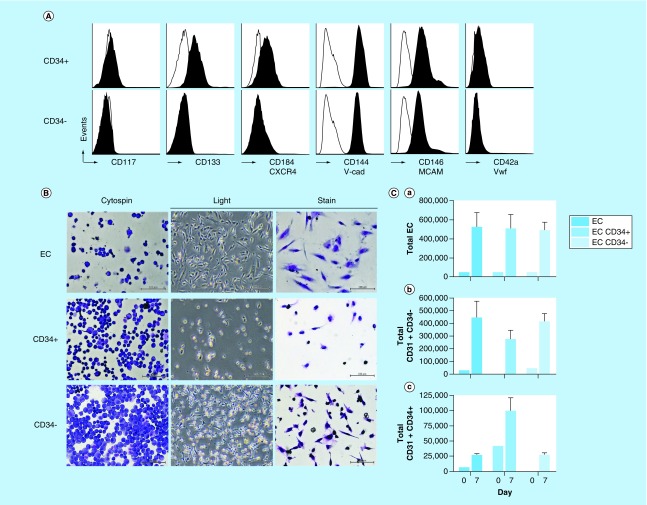

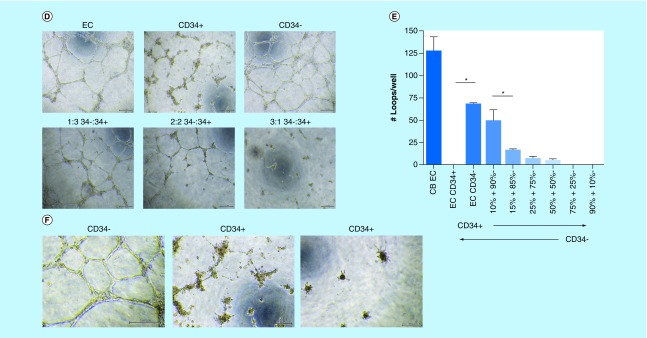
**Characteristics of cord blood ECFC/EC CD34^+^CD31^+^ and CD34^+^CD31^-^ subpopulations.** **(A)** CB ECs were cultured for 7 days in EndoGo and CD34^+^CD31^+^ and CD34^+^CD31^-^ were immunophenotyped for various cell surface markers. Open bar represents isotype. Black bar represents sample. Plots are representative of three experiments. **(B)** Morphological images of whole EC CB or CD34^+^CD31^+^ or CD34^+^CD31^-^ subpopulations. Left column obtained via cytospin followed by fixation and stain with Hema3. Right Column obtained after 24-h cell culture then fixed and stained with Hema3. (magnification, 4×). **(C)**. Growth characteristics of CB EC and EC subpopulations after seven days including total cell number **(a)**, total CD31^+^CD34^-^ **(b)** and total CD31^+^CD34^+^ **(c)**. **(D)** Matrigel assay of CB ECs and EC subpopulations. Population obtained through sorting and ratios combined prior to Matrigel seeding. **(E)** Graph depicts number of completed loops/well of each sample. Graph depicts mean ± standard error of the mean (*p < 0.05), n = 3. **(F)** Microscopic images of single CD34^-^ or CD34^+^ cells in Matrigel. CB: Cord blood; EC: Epithelial cell.

Since EndoGo specifically expanded the CB EC CD34^+^ population compared with ECM, we examined the CD34^+^ and CD34^-^ subpopulations for phenotypic, morphologic or functional differences. In Figure 2A, we used flow cytometry to phenotype for CD117 (*KIT*), CD133 (*PROM1*), CD184 (*CXCR4*), CD144 (*CDH5*), CD146 (*MCAM*) and CD42a (*GPIX*), all of which are markers used to identify ECs obtained from various blood sources. Both the CD34^+^ and CD34^-^ EC subpopulations were CD144^+^, CD146^+^ and CD42a^-^. However, CD133 and CD117 expression was detected on CD34^+^ ECs while not present on CD34^-^ ECs consistent with a progenitor-like population. Additionally, CD34^+^ ECs also expressed elevated levels of CD184, typically found on homing and niche-attached populations.

#### Morphology, growth characteristics & function of EndoGo expanded CB EC subpopulations

We next performed a cytospin analysis on ECFC/EC subpopulations to examine for morphological differences between the CD34^+^ and CD34^-^ CB EC subpopulations cultured in EndoGo. From Figure 2B (left column), the CD34^-^ EC subpopulation had a slightly larger cytoplasm to nucleus ratio (2:1), suggestive of a more mature subpopulation. The CD34^+^ EC subpopulation was similar in ratio (1:1). Figure 2B (right column) demonstrates the morphology of each population during *in vitro* growth and attachment (24 h). The CD34^-^ EC subpopulation demonstrated more fibroblast-like characteristics with cellular elongation and branching while the CD34^+^ EC subpopulation did not branch. Together, the smaller sized, larger nucleus:cytoplasm ratio and nonbranching CD34^+^ EC subpopulation is suggestive of a less mature, more progenitor-like population.

To examine for proliferative potential of each EC subpopulation, we sorted them and cultured in EndoGo (Figure 2C). After 7 days, both the CD34^+^ and CD34^-^ EC subpopulations had similar TNC when compared with the unsorted population (EC, 0.52 ± 0.16 × 10^6^; CD34^+^, 0.5 ± 0.16 × 10^6^; CD34^-^, 0.48 ± 0.09 × 10^6^) (Figure 2Ca). However, the isolated CD34^+^ culture continued to expand total CD34^+^ cells while the CD34- EC culture had minimal detection similar to the unsorted EC control (EC: 0.26 ± 0.04 x 10^5^; CD34^-^: 0.26 ± 0.05 × 10^5^; CD34^+^: 0.99 ± 0.22 × 10^5^). This same experiment performed with ECM could not be quantified as CD34^+^ cells do not expand in cultures (data not shown). Notably, both CD34^+^CD31^+^ and CD34^-^CD31^+^ ECs were expanded in the CD34^+^ isolated culture suggesting that lineage differentiation indeed begins with CD34^+^CD31^+^ cell phenotypes.

We next tested for functional differences between the CD34^+^ and CD34^-^ ECFC/EC subpopulations via tube formation in Matrigel (Figure 2D). Although robust tube formation occurred with the unsorted ECs, as expected, tube formation only occurred with the CD34^-^ subpopulation and was absent with CD34^+^ ECs (Figure 2D, top row). Most unique was the lack of Matrigel loop formation with increasing numbers of CD34^+^ cells. Upon addition of increasing ratios of CD34^+^ cells into CD34^-^ Matrigel cultures, tube formation was inhibited (Figure 2D, bottom row). Quantitatively, CD34^-^ matrigel assays contained more total loops per well, 63 ± 1, than CD34^+^ matrigel assays (0) with decreasing loop formation as more CD34^+^ cells were added to assay (Figure 2F). It is unclear why unsorted CB ECs formed more tubes in assay than the CD34^-^ population (126 ± 2), though sorting or selection may interfere initially with optimal tube formation.

EC sprouting begins the process of angiogenesis through growth factor signaling and cell activation. Upon closer examination of each subpopulation, CD34^+^ ECs produced enhanced sprouting while CD34^-^ ECs did not form sprouts. Together, this suggests that while tube formation is promoted by CD34^-^ ECs, initiation of angiogenesis is promoted by CD34^+^ cells. Enhanced microscopic inspection revealed significant sprouting of the individual cells in the CD34^+^ Matrigel culture while not present in the CD34^-^ Matrigel culture (Figure 2F).

### EndoGo expands CB EPCs

#### Identification of the CB EPC

**Figure 3.**
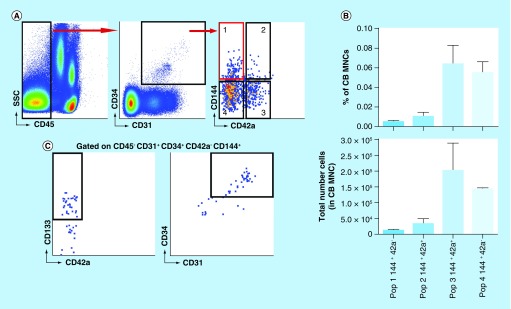

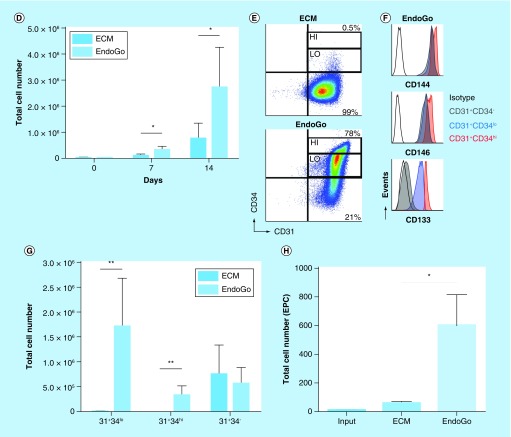
**Isolation and expansion of the cord blood EPC with EndoGo.** **(A)** Representative flow plots demonstrating gating for the CB EPC. **(B)** Top panel: % of CB EPC subpopulation in CB MNC. Bottom panel: total number CB EPC subpopulation in CB MNC. Gated off CD45^-^CD34^+^CD31^+^. Graph depicts mean ± SEM, n = 3. **(C)** CD133 expression (left panel) and backgated CD34/CD31 phenotype of the CB EPC (right panel). **(D)** Total EC after sorting CB EPC through expansion of P0 CB EPC colony growth in ECM or EndoGo. Graph depicts mean ± SEM (*p < 0.05), n = 3. **(E)** Flow cytometric phenotype of day 14 expanded EPCs. **(F)** CD144 (top), CD146 (middle) and CD133 (bottom) expression on either isotype (black line), CD31^+^CD34^-^ (gray-shaded), CD31^+^CD34^lo^ (blue-shaded) or CD31^+^CD34^hi^ (red-shaded) from EndoGo day 14 expansion. **(G)** Quantitative analysis of CD31^+^CD34^lo^, CD31^+^CD34^hi^ and CD31^+^CD34^-^ subpopulations in CB EPCs expanded in either ECM or EndoGo. **(H)** Quantitative analysis of the number of EPCs in CB-derived EC lines. Graph depicts mean ± SEM (*p < 0.05), n = 5; **p < 0.01 CB: Cord blood; EC: Epithelial cell; ECM: Endothelial cell media; MNC: Mononuclear cell; SEM: Standard error of the mean.

Although EndoGo significantly expanded CB ECFCs/ECs compared with a standard EC culture medium in *ex vivo* culturing, our results specifically focused on expansion of the CD34^+^ EC subpopulation. To determine if EndoGo could uniquely expand the CB EPC *in vitro*, we first sorted CB MNCs into CD45^-^CD34^+^CD31^+^ and CD45^-^CD34^+^CD31^-^ populations. After 3 weeks, ECFC/EC cell populations were only generated from the CD45^-^CD34^+^CD31^+^ population, with an EC phenotype of CD31 (data not shown). However, to further clarify the EPC phenotype, we examined for CD144, CD42a and CD146 which were expressed on the EndoGo expanded CB ECs. CD146 was minimally detected (<0.3%) on CD45^-^ CB MNCs as observed with flow cytometry and not included (data not shown). We then sorted four different populations from the CD45^-^CD34^+^CD31^+^ gate using CD42a and CD144 and initiated into culture: CD45^-^CD34^+^CD31^+^CD144^+^CD42a^-^; CD45^-^CD34^+^CD31^+^CD144^+^CD42a^+^; CD45^-^CD34^+^CD31^+^CD144^-^CD42a^+^ and CD45^-^CD34^+^CD31^+^CD144^-^CD42a^-^ (Figure 3A). Total number analysis of each sorted population revealed the CD45^-^CD34^+^CD31^+^CD144^+^CD42a^-^ (Population 1) as an infrequent cell population within CB MNCs (7073 ± 767 per CB, 0.005 ± 0.002%; Figure 3B); however, ECFC/EC growth initiated only from this population after 3 weeks. The remaining cultures demonstrated early adherent, fibroblastic-type cells before eventually dying in culture (data not shown). Flow cytometric analysis further revealed Population 1 had significant CD133 expression (62.1 ± 9.1%), a feature that is supported by establishment of EC lines via CD133 selection (Figure 3C, Left Panel). Backgating analysis also revealed that Population 1 was contained largely in the CD34^hi^CD31^hi^ phenotype (Figure 3C, Right Panel). Herein, the CB EPC is defined as CD45^-^CD34^hi^CD31^hi^CD144^+^CD42a^-^CD133^+^.

#### EndoGo expansion of the CB EPC

After establishing early CB EPC colonies, we separated the cells into either ECM or EndoGo to examine whether *ex vivo* media can expand freshly isolated CB EPC (Figure 3D–G). After 7 days, EndoGo significantly expanded total CB ECs 3× greater than ECM (0.33 ± 0.14 × 10^6^ and 0.11 ± 0.047 × 10^6^, respectively); however, robust expansion of CB ECs occurred in EndoGo after 14 days (ECM, 0.76 ± 0.491 × 10^6^ and EndoGo, 2.72 ± 1.01 × 10^6^) (Figure 3D). This robust expansion continued through Day 21 (ECM, 5.03 × 10^6^ and EndoGo, 49.0 × 10^6^), though expansion is only theoretical due to splitting culture at Day 14 (data not shown). Figure 3E demonstrates the flow cytometric phenotype of ECM or EndoGo expanded EPCs after 14 days. Although the CD31^+^CD34^+^ population was almost negligible in ECM (0.5%), EndoGo maintained significantly more expression (78%). Further EndoGo expanded a CD31^hi^CD34^hi^ population that expressed elevated levels of CD133 compared with the CD31^+^CD34^lo^ or the CD31^+^CD34^-^ population (Figure 3F). Similar expression levels of CD144 and CD146 in all populations were observed suggesting EndoGo targeting CD133 and CD34 expression on the EPC. In 14-day expansion, EndoGo significantly increased the CD31^+^CD34^hi^ (EPC) population compared with ECM (Input: 3536 ± 384; ECM: 15 ± 10, EndoGo: 0.33 ± 0.1 × 10^6^ (93-fold) (Figure 3G). The CD31^+^CD34^lo^ population was also significantly expanded in EndoGo compared with ECM (ECM: 3867 ± 1000; EndoG: 1.7 ± 0.8 × 10^6^) (Figure 3G).

To further confirm the effectiveness of CB EPC expansion by EndoGo, we used the CD45^-^CD34^+^ EC lines generated from multiple CB sources to expand cells and phenotype for the EPC (Figure 3F). Importantly, EndoGo uniquely expanded the CB EPC compared with ECM after only seven days in culture (Input: 11 ± 0; ECM: 59 ± 12 [5-fold]; EndoGo: 603 ± 215 [54-fold]). This further demonstrates the effective expansion properties of EndoGo on CB ECs *in vitro* and more specifically in targeting expansion of the CB EPC.

## Discussion

*Ex vivo* expansion of ECs could greatly benefit cell therapeutic treatment of many different diseases, including ischemia, stroke and diabetes. Currently, an expansion culture has not been developed that promotes efficient expansion of CB ECs or even CB EPCs at clinically relevant numbers. Here, we demonstrate significant expansion of CB ECFCs/ECs and EPCs using EndoGo medium, with specific addition of HPL, a fact recently established in long-term cultures of PB ECs [30]. Various reports have suggested clinical-scale expansion of ECs in respective cultures but these efforts were based solely on theoretical calculations and not clinical scale experimentation [22,23]. Numerous studies have developed *ex vivo* cultures to expand EPCs (reviewed in [31]). Senegaglia *et al*. demonstrated 70-fold expansion of CB EPCs (6.23 × 10^6^ total) generated from CD133+ CB cells over 60 days [32]. Lippross *et al*. reported a 117.6-fold increase in EPCs generated from BM CD34^+^ (2.94 × 10^6^ total) or 147.9-fold increase in EPCs from BM CD133+ (3.7 × 10^6^ total) over 30 days [33]. Masuda *et al*. induced a 52.9-fold increase (0.5 × 10^6^ total) in biologically functional EPCs (based on colony formation) over 7 days [34]. In long-term cultures (28 days, Day 0; EC: 25000, EC 34+: 1560) of CB EC cell lines with weekly passages, EndoGo could induce 679-fold expansion of total ECs (17 × 10^6^ total), while ECM promoted only 136-fold expansion (3.4 x 10^6^ total) (data not shown). Importantly, expansion of the CD34^+^ fraction in 28-day cultures with EndoGo compared with ECM was significantly higher (EndoGo: 2478-fold [3.9 × 10^6^ total]; ECM: 88-fold [0.13 x 10^6^ total]). This is significant considering reports citing EPC expansion would demonstrate a loss of CD34 or CD133 expression through long-term cultures [23,32,34]. Future work is being explored to attempt actual clinical scale expansion of CB ECs with EndoGo (data not shown).

EndoGo induced expansion of two separate populations within CB ECs that phenotypically, morphologically and functionally were different, CD34^+^CD31^+^ and CD34^+^CD31^-^. One recent study has demonstrated induction of CD34^+^ expression on CD34^-^ PB ECs in confluent cultures [35]. We seeded CD31^+^CD34^-^ CB ECs at higher concentrations and observed some re-expression of CD34 on the CD34^-^ cells, specifically when cultures became confluent (data not shown). Conversely in separate confluent cultures, media changes performed every other day inhibited CB EC growth (data not shown). This is suggestive of a more complicated autocrine and paracrine signaling of subpopulations within CB EC *ex vivo* cultures that needs to be discovered.

Our studies identified the CD34^+^ fraction as a more progenitor-like population: flow phenotype of CD117, CD133, CD34 and CXCR4; morphology with higher nuclear:cytoplasmic ratio and ability to derive CB ECs from CD34^+^ fraction. Earlier studies have also reported expression of CD117 and CD184 (CXCR4) [16] on EPCs and their importance for EC homing and initiation of tissue repair [36,37]. Clinical trials targeting CXCR4 have been used to mobilize EPCs for tissue repair during myocardial infarct and ischemia [38,39]. Various studies have used these markers to phenotype stem and progenitor cell populations of HSCs [40] and cancer stem cells [41]. This is supportive of the notion of EndoGo expansion of the CB EC progenitor cell for use in cell therapy protocols.

The inability of the cultured CD34+ CB ECs to form tubes in the Matrigel assay while displaying increased sprouting depicts a population that may home to injured sites and initiate angiogenesis. We attempted long-term culturing of CD34^+^ cells on Matrigel but cell viability decreased and cells never formed tubes (data not shown). Although these results are similar to Ferreras *et al*. [42], an opposite report by Tasev *et al*. demonstrated that the CD34^+^ EC fraction could indeed form tubes within a matrix assay [35]. This discrepancy may be due to the different EC culture conditions since both groups isolated ECs from PB samples. However, the use of different sources cannot be ruled out as phenotypes could vary between products, which would require further testing.

The exact phenotype of the CB EPC is currently unknown, although many reports have successfully identified unique markers [12,43]. Conflicting reports demonstrating EPC isolation from both hematopoietic (CD45^+^ and nonhematopoietic (CD45^-^) sources as well as opposing markers (CD34^-^) cause inconsistencies in EC biology discussions [12,25,44,45]. Expression of monocyte-macrophage markers on *in vitro* ECs have been identified further suggesting a hematopoietic link to the EPCs [45,46]. We did not detect CD14 expression on *in vitro* cultured CB ECs throughout culturing and efforts to isolate ECs from the CD45^+^ CB MNC population were also not successful (data not shown). Other groups have attempted to define EPCs from PB and BM using CD144, CD42a and CD146, although methods of isolation and culturing were varied [47,48]. Variances in isolation techniques and culture regimens could explain these inconsistencies; more specifically, these markers were not applied in a specific panel to isolate and promote EC growth. Attempts to provide a consensus nomenclature have demonstrated a largely unknown phenotype to clarify or determine specific subsets of ECs and for specific lineage tracing [49]. We explicitly utilized multimarker cell sorting to isolate various subpopulations within CB and seeded these subpopulations into identical cultures to uniquely identify the EPC phenotype and avoid confusion. It is also interesting to note that the CB EPC we identify, derived from the CD45^-^CD34^+^CD31^+^CD144^+^CD42a^-^ population, exhibited higher differential expression of CD34^+^ and CD31^+^. This demonstrates the fluid nature of cell surface markers and requires caution when using markers as absolutes when attempting to identify cell populations through flow cytometry. Although the detection of CD133 on this population validates other studies that isolated ECs using this marker [14,16,18,44], expression of CD133 is not unique to EPCs as expression is also observed on HSCs [18,40]. Furthermore, though CD133 expression was only observed in the CD42a^-^ fraction, some expression was detected on the CD144^-^ population establishing heterogeneity of CD133 on EC subpopulations (data not shown).

To our knowledge, this is the first report demonstrating multiple markers on a single population where CB ECs can be derived. Our CB EPC phenotype represents 0.003–0.004% of total CB MNCs which makes detection and thus assaying difficult. However, upon early proliferation of CB-derived ECs, the effectiveness of EndoGo expansion compared with ECM on the CB EPC enables in-depth study of this population (Figure 3). Future work needs to determine the effectiveness of EndoGo compared with other commercially available EC mediums, specifically regarding the ability to expand large, clinical-scale numbers of total ECFC/ECs, EC progenitor cells and possibly heterogeneous subpopulations that might impact effectiveness of specific targeted cell therapy. Specific expansion of the CB EPC we identified is critical given the heterogeneic phenotype demonstrated in various EC expansion studies. Further, we established expansion of EPCs using the phenotype at the end of culture, which is lacking in many publications suggesting EPC expansion. We discovered that EndoGo could specifically expand the unique phenotype of the CB EPC that we used to freshly isolate from CB, maintaining this population 2–3 weeks *ex vivo*.

Although mobilization studies of ECs have been reported for treatment of ischemia and stroke, success is limited due to the age and disease status of patients along with potential for further damage to patient tissues from mobilization of other cell populations [50,51]. *Ex vivo* expansion provides an exciting alternative to generating EC or EPCs for cellular therapy. In this study, EndoGo demonstrates a unique capability of significantly expanding total CB ECFC/EC numbers and promoting the expansion of CB EPCs. Although more studies are needed to effectively determine if EndoGo could produce large-scale, clinically relevant numbers of ECs leading to more effective therapies, EndoGo provides an excellent medium to expand ECFC/EC cell subpopulations, which could be useful in determining whether certain populations are needed to promote specific angiogenesis of arterial, venous or capillary vasculature. Further work needs to address whether these *ex vivo* expanded CB ECFCs/ECs can be utilized in treating disease models, whether in animal models or clinical-scale translatable expansions into clinical trials. Study and characterization of ECs from different sources also needs to be clarified to better understand EC biology and to develop protocols for cellular therapy.

## Conclusion & future perspective

Although further studies are needed to determine how efficiently EndoGo can expand endothelial cells, this study is an important beginning step in developing an effective protocol to expand endothelial cells for clinical translation. This study also builds on the developing groundwork regarding the CB EPC that EndoGo targets for proliferation and expansion. Better understanding of specific subpopulations of endothelial cells may contribute to the improvement of EC expansion protocols for clinical cell therapy. Further detailed studies into the EPC and lineage progression could enable development of better *ex vivo* expansion protocols to promote larger clinical-scale numbers for infusion into patients undergoing cell therapy.

**Summary points**

- Revascularization of tissues is essential for clinical therapies.
- Large clinical-scale numbers are needed for endothelial cell therapy.
- Insufficient endothelial cell numbers exist in cord blood (CB) products and *ex vivo* expansion provides an approach to overcome that deficiency.
- EndoGo XF significantly expands CB endothelial cells.
- CD34^+^ CB endothelial cells can be expanded using EndoGo XF.
- *Ex vivo* expanded CB CD34^+^ endothelial cells demonstrate less fibroblast-characteristics and lack of tube formation *in vitro* compared with CD34^-^ endothelial cells, suggestive of an angiogenic initiating cell.
- EndoGo XF can significantly expand the CB endothelial progenitor cell.
